# Aspirin induces apoptosis *in vitro* and inhibits tumor growth of human hepatocellular carcinoma cells in a nude mouse xenograft model

**DOI:** 10.3892/ijo.2011.1304

**Published:** 2011-12-15

**Authors:** MOHAMMAD AKBAR HOSSAIN, DONG HWAN KIM, JUNG YOON JANG, YONG JUNG KANG, JEONG-HYUN YOON, JEON-OK MOON, HAE YOUNG CHUNG, GI-YOUNG KIM, YUNG HYUN CHOI, BRYAN L. COPPLE, NAM DEUK KIM

**Affiliations:** 1 Division of Pharmacy, College of Pharmacy, Molecular Inflammation Research Center for Aging Intervention (MRCA), Pusan National University, Busan 609-735; 2 Faculty of Applied Marine Science, Cheju National University, Jeju 690-756; 3 Department of Biomaterial Control (BK21 Program), Dongeui University Graduate School, Busan 614-052, Republic of Korea; 4 Department of Pharmacology and Toxicology, Michigan State University, East Lansing, MI 48824, USA

**Keywords:** aspirin, hepatocellular carcinoma, apoptosis, xenograft model

## Abstract

Nonsteroidal anti-inflammatory drugs (NSAIDs) are known to induce apoptosis in a variety of cancer cells, including colon, prostate, breast and leukemia. Among them, aspirin, a classical NSAID, shows promise in cancer therapy in certain types of cancers. We hypothesized that aspirin might affect the growth of liver cancer cells since liver is the principal site for aspirin metabolism. Therefore, we investigated the effects of aspirin on the HepG2 human hepatocellular carcinoma cell line *in vitro* and the HepG2 cell xenograft model in BALB/c nude mice. We found that treatment with aspirin inhibited cell growth and induced apoptosis involving both extrinsic and intrinsic pathways as measured by DNA ladder formation, alteration in the Bax/Bcl-2 ratio, activation of the caspase activities and related protein expressions. *In vivo* antitumor activity assay also showed that aspirin resulted in significant tumor growth inhibition compared to the control. Oral administration of aspirin (100 mg/kg/day) caused a significant reduction in the growth of HepG2 tumors in nude mice. These findings suggest that aspirin may be used as a promising anticancer agent against liver cancer.

## Introduction

Hepatocellular carcinoma (HCC) represents the fifth most common malignancy and the third most frequent cause of cancer death around the world (1). Hepatitis B and C virus infections, exposure to aflatoxin, and excessive intake of alcohol have been identified as major risk factors of HCC. Surgery is the most effective option, but unfortunately the majority of patients with HCC are not amenable to surgery at the time of diagnosis. Presently, one of the main approaches in treating inoperable HCC is to use cytotoxic chemotherapy, but sometimes HCC is less sensitive to or becomes resistant to anticancer drugs after consecutive treatments; most tests failed to find a therapy which can produce a response rate >25% among hepatoma patients (2,3). Despite recent progress in diagnosis and treatment, HCC is still diagnosed at an advanced stage where prognosis is poor. Important efforts should therefore be directed toward developing effective and less toxic chemotherapeutic strategies.

Aspirin (acetylsalicylic acid) is the best-known salicylate and belongs to the pharmacologic category of the nonsteroidal anti-inflammatory drugs (NSAIDs). Despite wide use being made for more than 100 years, clinical uses of aspirin have been changed over time and knowledge on its mechanisms of action and therapeutic entities continually evolve. During the first fifty years since it was developed, aspirin was primarily used as an analgesic, anti-pyretic, and anti-inflammatory agent based on its main mechanism of action of inhibiting prostaglandin synthesis. However, currently aspirin is more commonly used as an anti-thrombotic agent, in primary and secondary prevention of thromboembolic events. The suggestion that aspirin could be of benefit against cancer initially arose from the observation that tumor metastases were reduced in rats with thrombocytopenia (4–6). Subsequently, prostaglandin concentration proved to be raised in rat colorectal tumor tissues (7,8), which strengthened the expectation that anticancer benefit might be gained through the inhibition of cyclooxygenase (COX).

One obvious molecular target for aspirin is COX-2 which is the enzyme highly and rapidly induced in response to mediators of inflammation, growth factors, cytokines, or endotoxins, and is involved in cell proliferation and tumor promotion (9). This is supported by the fact that aspirin can decrease the production of potentially neoplastic prostaglandins produced from COX-2-mediated catalysis of arachidonic acid (10). The carcinogenic contribution of prostaglandins has generated much interest; their deleterious effects include promotion of cell survival, stimulation of cell proliferation, and promotion of angiogenesis (11,12). These effects can also enhance cancer spread and thus underscore the cancer fighting potential of aspirin.

However, the anticancer effect of aspirin and NSAIDs cannot be solely explained by the inhibition of prostaglandin synthesis, since several NSAIDs have antiproliferative effects in cells that have no COX activity. High doses of aspirin have been reported to induce apoptosis through COX-independent mechanisms, by regulating several different targets (13), such as *15-LOX-1* (14), a proapoptopic gene *Par-4* (15), and an antiapoptopic gene *Bcl-X_L_* (16). Additionally, NSAIDs including aspirin also induce apoptosis by means of the activation of caspases (17,18), the activation of p38 MAP kinase (19), release of mitochondrial cytochrome *c* (18–20), and activation of the ceramide pathway (21). These effects might not be universal to all cell types, however, and the dose range of aspirin needed in such COX-independent pathways could be higher than that for the inhibition of COX-2 (22). Other mechanisms contributing to the potential anticancer effects of aspirin could be attributed to the upregulation of tumor suppressor gene, such as *Bax*, and the downregulation of antiapoptotic gene, such as *Bcl-2* (23). Apoptosis (programmed cell death) has been recognized as an important physiological event in the development and pharmacology of anticancer agents and cancer therapies (24,25). In recent years, the regulation of apoptosis has become an area of extensive study in cancer research as the life span of both normal and cancer cells within a living system is regarded to be significantly affected by the rate of apoptosis (26,27).

Raza *et al* (18) recently demonstrated that aspirin treatment (5–10 μmol/ml) induced oxidative stress, cell cycle arrest in the G0/G1 phase, apoptosis, and mitochondrial dysfunction in HepG2 cells *in vitro*. In this study, we evaluated the effects of aspirin on apoptosis induction in human hepatocellular carcinoma cell line *in vitro* and antitumor activity in HepG2 cell xenograft of nude mouse model.

## Materials and methods

### Chemicals

Aspirin was purchased from Sigma-Aldrich Co. (St. Louis, MO). Aspirin was freshly prepared before each experiment and solubilized as described elsewhere (28). All other chemicals and reagents were from standard commercial sources and of the highest purity.

### Cell culture and cell viability assay

The human hepatocellular carcinoma cell line HepG2 (wild-type p53; Rb-positive; Ras-mutated; and HBV-negative) cells were purchased from the American Type Culture Collection (Manassas, VA), and maintained at 37°C in a humidified condition of 95% air and 5% CO_2_ in DMEM (Gibco-BRL, Gaithersburg, MD) supplemented with 10% heat inactivated fetal bovine serum (FBS), 2 mM glutamine, 100 U/ml penicillin, and 100 μg/ml streptomycin. Cell proliferation was assessed using MTT [3-(4,5-dimethylthiazol-2-yl)-2,5-diphenyltetrazoliumbromide, Sigma-Aldrich Co.] assay, as described before (29), which is based on the conversion of MTT to MTT-formazan by mitochondrial enzymes.

### DNA fragmentation assay

DNA fragmentation was performed as described previously (30). Briefly, after treatment with aspirin, cells were rinsed twice in cold PBS and resuspended in lysis buffer [5 mM Tris-HCl (pH 7.5), 5 mM ethylene diamine tetraacetic acid and 0.5% Triton X-100] at 4°C for 30 min. After centrifugation at 27,000 × g for 15 min, the supernatant was treated with RNase, followed by proteinase K digestion, phenol/chloroform/isoamyl alcohol (25:24:1, v/v/v) extraction and isopropanol precipitation. DNA was separated through a 1.5% agarose gel, and stained with 0.1 μg/ml ethidium bromide (EtBr, Sigma-Aldrich Co.), and was visualized by ultraviolet light source.

### Caspase activity assay

Activities of caspase-3, -8 and -9 were determined using the corresponding caspase activity detection kits (R&D Systems, Minneapolis, MN) as described by the manufacturers. Briefly, cells were harvested and cold lysis buffer was added, and then incubated on ice for 10 min and centrifuge for 1 min in a microcentrifuge (10,000 × g). The supernatant was transferred to a fresh tube and protein concentration was determined using a standard colorimetric assay (Bio-Rad Laboratories, Hercules, CA). The protein concentration of each sample was adjusted to 200 μg per 50 μl of cell lysate using chilled cell lysis buffer. Then 50 μl of 2X reaction buffer was added and 5 μl of substrates of DEVD-*p*NA (for caspase-3), IETD-*p*NA (for caspase-8), and LEHD-*p*NA (for caspase-9), respectively. Samples were incubated at 37°C for 2 h and the enzyme-catalyzed release of *p*NA was quantified at 405 nm using a microtiter plate reader. The values of aspirin treated samples were normalized to the untreated controls, allowing determination of the fold increase in caspase activity.

### Gel electrophoresis and western blot analysis

The cells were harvested, lysed, and protein concentrations were quantified using the Bio-Rad protein assay (Bio-Rad Laboratories), following the procedure described by the manufacturer. Western blot analysis was performed as described previously (31). Briefly, an equal amount of protein was subjected to electrophoresis on SDS-polyacrylamide gels and transferred to nitrocellulose membranes (Schleicher & Schuell, Keene, NH) by immunoblotting. Blots were probed with the desired antibodies for overnight, incubated with diluted enzyme-linked secondary antibodies and then visualized by the enhanced chemiluminescence (ECL) according to the recommended procedure (Amersham Corp., Arlington Heights, IL). The primary antibodies were purchased from Santa Cruz Biotechnology Inc. (Santa Cruz, CA). Peroxidase-labeled goat anti-rabbit and goat anti-mouse immunoglobulin were purchased from Santa Cruz Biotechnology Inc. and Amersham.

### In vivo xenograft model

Six-week-old male BALB/c nude mice (obtained from Japan SLC, Inc., Japan) were used for *in vivo* animal experiments. The animals were housed in constant laboratory conditions of a 12-h light/dark cycle and specific pathogen-free conditions and fed with water and food *ad libitum*. The animal protocol used in this study has been reviewed by the Pusan National University-Institutional Animal Care and Use Committee (PNU-IACUC, Busan, Korea) on their ethical procedures and scientific care, and it has been approved. For xenograft study, mice were inoculated subcutaneously into the right-back with 7.5×10^6^ HepG2 cells in 200 μl PBS and Matrigel (1:1). The mice were randomly assigned into two groups of 6 each; one group received oral aspirin, suspended in 0.5% sodium carboxymethyl cellulose (Na-CMC), at 100 mg/kg/day; the other group was used as the control that received same amount of Na-CMC in water orally. The body weight and tumor volume [(major axis) × (minor axis)^2^ × 1/2] of every mouse were monitored biweekly after 4 weeks up to the end of the experiment (7 weeks).

### Histopathological findings

Xenograft tumor and stomach tissue samples were fixed in 10% neutral buffered formalin, dehydrated, and embedded in paraffin. Samples were subsequently sectioned at 5 μm thickness, and stained with hematoxylin and eosin (H&E) for histopathology.

### Statistical analysis

The data are presented as means ± SEM of three independent determinations. ANOVA was conducted to analyze significant differences among all groups. Other statistical analyses were carried out by Student's t-test. p<0.05 and p<0.01 were considered to be significant.

## Results

### Aspirin reduced the viability of cells

**Figure 1:**
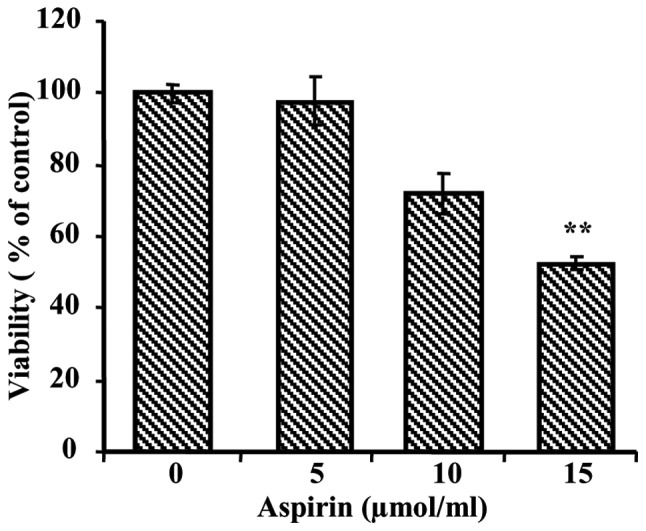
Effect of aspirin on the viability of HepG2 cells. The cells were treated with different concentrations of aspirin for 24 h, and then percentage of cell survival was determined using the MTT assay. Results are expressed as percentage of the vehicle treated control ± SEM of three separate experiments. (^**^p<0.01 vs. untreated control cells).

To evaluate the growth inhibiting effects of aspirin on human hepatocellular carcinoma cell line, we initially performed an MTT assay. The MTT assay showed increasing cytotoxicity of aspirin in HepG2 cells after 24 h of treatment. As shown in Fig. 1, cell viability was significantly decreased by treatment of aspirin in a dose-dependent manner. The dose required for half-maximal inhibition (IC_50_) of HepG2 cell growth was ~15 μmol/ml.

### Aspirin induces apoptosis

**Figure 2:**
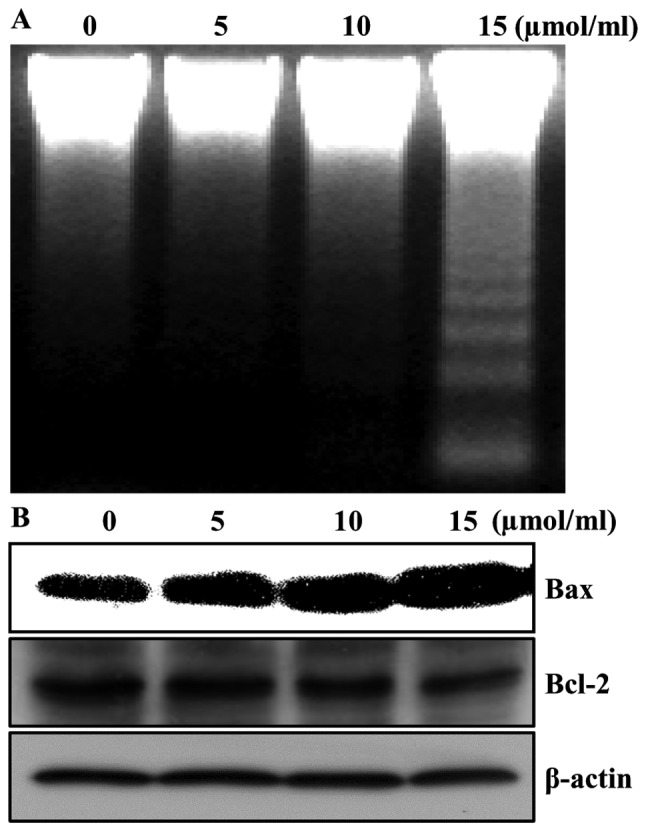
Induction of apoptosis in HepG2 cells by aspirin. Cells were incubated with different concentrations of aspirin for 24 h. (A) DNA fragmentation was detected by 1.5% agarose gel containing EtBr. (B) Bax and Bcl-2 protein expressions in HepG2 cells treated with aspirin were analyzed by western blot analysis using anti-Bax and anti-Bcl-2 antibodies and ECL detection. Actin was used as an internal control. A representative blot is shown from three independent experiments.

In order to determine whether the growth inhibition by aspirin was associated with the induction of apoptosis in HepG2 cells, we analyzed DNA fragmentation which is a hallmark of apoptosis. Following agarose gel electrophoresis of the cells treated with 5, 10 and 15 μmol/ml of aspirin for 24 h, a typical ladder pattern of internucleosomal fragmentation was observed (Fig. 2A). Since apoptosis might be regulated by the alteration in the ratio of Bax/Bcl-2 family protein expression, we tested whether aspirin-induced apoptosis was accompanied by the change of the expression levels of Bax and Bcl-2. The results from western blotting showed that treatment with aspirin resulted in the up-regulation of Bax and slightly decreased in Bcl-2 expression in cells, suggesting aspirin treatment alters the Bax/Bcl-2 ratio in HepG2 cells (Fig. 2B). Taken together, these results implied that the cytotoxic effects observed in response to aspirin were most likely to be associated with the induction of apoptotic cell death.

### Aspirin induces apoptosis through extrinsic and intrinsic pathways and caspase cascade

**Figure 3:**
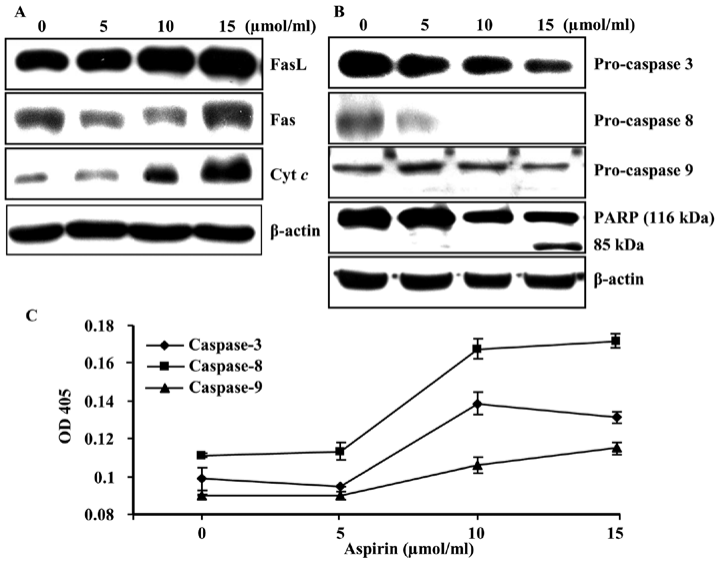
Aspirin induces the expression of (A) apoptotic related protein levels and (B) caspase cascade in HepG2 cells. After 24 h of incubation with aspirin, total cell lysates were prepared and immunoblotted. Western blots were detected with corresponding antibodies and ECL detection. Actin was used as an internal control. A representative blot is shown from three independent experiments. (C) Activation of caspase-3, -8, and -9 in HepG2 cells. Cells were treated with aspirin for 24 h at indicated concentration and then levels of caspase activity were measured. Data are expressed as mean ± SEM of three independent experiments.

To investigate the mechanisms whereby apoptosis is induced, we examined the death receptor and death receptor mediated proteins as well as lethal stimuli of mitochondria inside the cells using immunoblot analysis. A significant increase in the expression levels of FasL, Fas, and cytochrome *c* were observed (Fig. 3A). In addition, induction of procaspase-3, -8, and -9 and the subsequent proteolytic cleavage of poly(ADP-ribose) polymerase (PARP) were also observed in a dose-dependent manner (Fig. 3B). Moreover, aspirin treatment significantly increased caspase-3, -8, and -9 activities in a dose-dependent manner (Fig. 3C). These results indicate that aspirin treatment induces apoptotic death in HepG2 cells, at least in part through a caspase-dependent pathway.

### Aspirin administration reduces the growth of HepG2 xenografts

**Figure 4:**
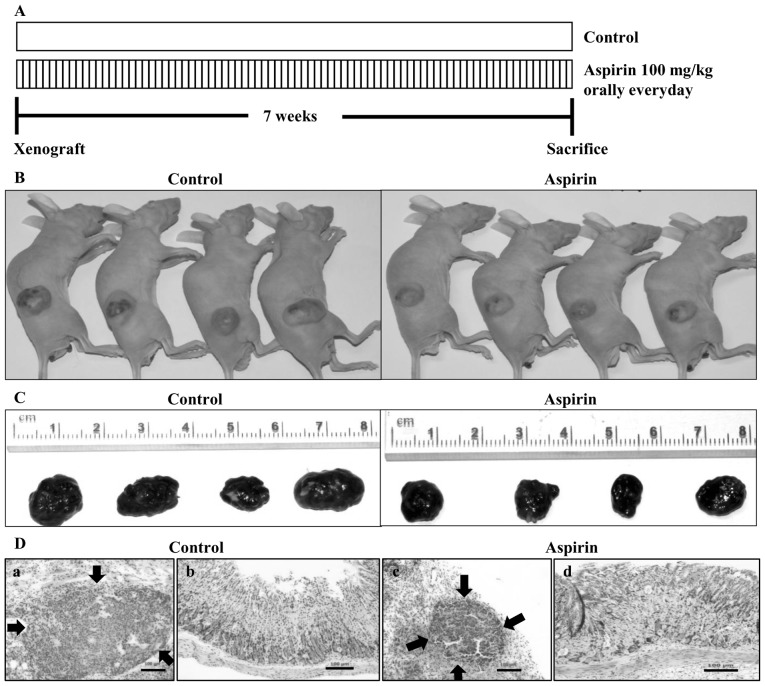
Aspirin inhibits growth of HepG2 cell xenograft in nude mice. (A) Experimental protocols in BALB/c nude mice. All mice were divided into two groups of 6 mice each, control and aspirin. Nude mice at 7 weeks of age were injected s.c. with 7.5×10^6^ HepG2 cells. After 1 day, mice were treated with aspirin at 100 mg/kg/dose or with 0.5% CMC in water as a vehicle control orally for the entire 7-week experimental period. (B) The mice with xenograft HepG2 cells of both groups. (C) Tumor images of both control and aspirin group. (D) Histopathological sections of the xenograft tumor (arrows) and stomach in nude mice of control group (a and b) and aspirin group (c and d), respectively. Representative images are shown from *in vivo* experiments. Bar, 100 μm.

Our *in vitro* observation suggested a potential role of aspirin in the treatment of liver cancer. Therefore, we examined the ability of aspirin to inhibit tumor growth of HepG2 cells in a nude mouse xenograft model. Aspirin was administered orally everyday at the dose of 100 mg/kg. After 7 weeks of treatment, mice were sacrificed and the tumors were collected (Fig. 4A). Image of tumors before (Fig. 4B) and after (C) necropsy showed that aspirin treatment resulted in shrinkage of tumor size. Histopathological sections of xenograft tumors in nude mice were invariably encapsulated by connective tissue and there was no histopathological difference except the size of tumors between control (Fig. 4D-a) and aspirin-treated mice (D-c). Histopathological examination of stomachs of control mice (Fig. 4D-b) and aspirin-treated mice (D-d) revealed that the aspirin dose (100 mg/kg) used in this study showed no degenerative changes in the stomach tissues compared to the control group.

**Figure 5:**
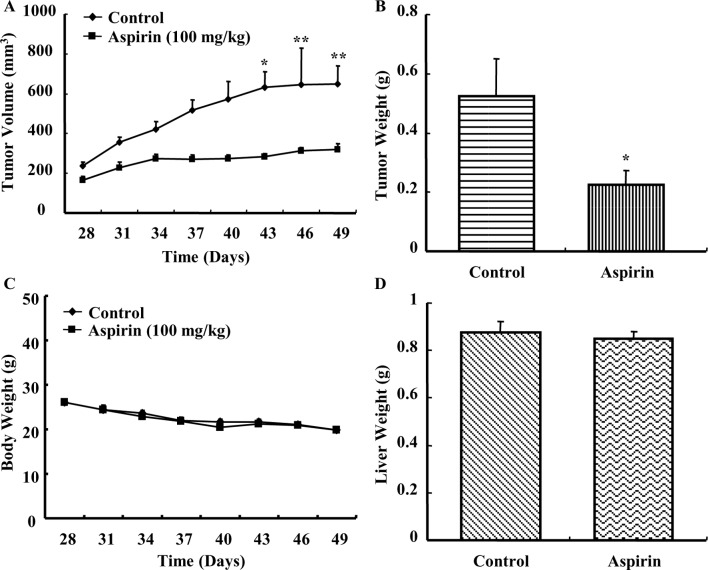
Aspirin inhibits xenograft HepG2 tumor growth. (A) Tumor growth curve. The volume of each tumor was measured twice a week after 4 weeks. The average tumor volume in vehicle-treated control mice and treated with aspirin is plotted. (^**^p<0.01 vs. vehicle-treated control group). (B) Final tumor weight at 7 weeks. (^*^p<0.05 vs. vehicle-treated control group). (C) Body weight changes during the experiment. (D) Liver weight at 7 weeks.

Aspirin treatment caused a time-dependent regression of HepG2 xenograft tumors in nude mice as compared to the control group (Fig. 5A). A considerable reduction in tumor weight (Fig. 5B) was observed in mice treated with aspirin. The average body weight of control or aspirin-treated mice did not vary significantly throughout the experiment (Fig. 5C). Additionally, the average liver weight of both control and aspirin-treated mice remained almost the same after the experiment (Fig. 5D). There were no observable signs of distress in aspirin-treated animals compared with control. These results indicated that aspirin reduced the growth of HepG2 xenografts in nude mice without causing any observable side effects.

## Discussion

The ability of aspirin to trigger apoptosis in cancer cells is well known and is consistent with the clinical and epidemiological evidence on its anticancer effects. The aim of the study was to investigate the anticancer activity of aspirin in HepG2 human hepatocellular carcinoma cells. This study revealed that aspirin had significant apoptotic activity against human hepatocellular carcinoma cell line in culture and suppressed growth of HepG2 cell xenograft tumors in nude mice.

Understanding of cell death signaling pathways is relevant to understanding cancer and to developing more effective anticancer agents. The induction of apoptosis by aspirin was confirmed by DNA fragmentation. The regulation of apoptosis is a complex process and involves a number of gene products including Bcl-2 family protein. It has been shown that the Bcl-2 family plays important regulatory roles in apoptosis, either as inhibitor or as activator. Thus, it has been suggested that the ratio between the level of pro-apoptotic Bax protein and that of the anti-apoptotic factor Bcl-2 protein determines how a cell responds to an apoptotic signal (16,32). In our study, there was a concentration-dependent increase of Bax protein levels in HepG2 cells treated with aspirin and the levels of Bcl-2 slightly decreased, and this consequently led to an increase in the ratio of Bax/Bcl-2 as we previously described (31).

The apoptotic signaling pathway that leads to caspase activation is subdivided into two major categories, extrinsic or intrinsic pathways. The anti-apoptotic Bcl-2 protein has been found to be associated with the mitochondrial membrane and to prevent both the loss of the mitochondrial membrane potential and the efflux of cytochrome *c* (33). Substantial evidence has been accumulated suggesting that release of cytochrome *c* from mitochondria is an important step in apoptosis. Once released in the cytosol, cytochrome *c* binds to Apaf-1 in a dATP/ATP-dependent manner, an event that triggers oligomerization of Apaf-1/cytochrome *c* in complexes that activate procaspase-9 (34). The ensuing recruitment and activation of caspase-9 result in activation of caspase-3, -6, and -7, which function as downstream effectors of the cell death program. Caspase-3 is an executioner caspase that also associated with death receptor pathway involving caspase-8 (33).

The extrinsic pathway is initiated by binding of the transmembrane death receptors with their specific ligands (Fas/FasL). Once activated, the intracellular domains of these receptors (DD) bind to the adaptor protein Fas-associated death domain (FADD) or TRADD (TNFR1-associated death domain protein) to form the death inducing complex (DISC) with recruitment of procaspase-8. Procaspase-8 is in turn proteolytically activated and serves as the ‘initiator’ caspase, further activating downstream effector proteins such as caspase-3, -6 and -7 to initiate cell degradation, and thereby causing inevitable apoptosis (35). Activation of caspases results in cleavage and inactivation of key cellular proteins, including the DNA repair enzyme PARP (36). Therefore, we evaluated the involvement of various caspases, death receptor with their ligand, cytochrome *c* and PARP during aspirin-mediated apoptosis in HepG2 cells. Our results implied that the involvement of caspase-3, -8, -9, FasL, Fas, cytochrome *c*, and PARP in execution of aspirin-induced apoptosis. Of note, we reported that oral administration of aspirin caused a considerable reduction in the growth of HepG2 cell xenograft tumors in nude mice. The dose used in this study (100 mg/kg/day) can be translated to a clinical dose of 520 mg for average body surface area or approximately one aspirin tablet taken for analgesic purposes in humans (37). There was no morbidity due to treatment, nor was there drastic variation in activity level or significant weight loss or gain between control and aspirin-treated animals indicating low toxicity of aspirin *in vivo*.

Experimental data presented herein showed that aspirin induced cell death and reduced growth of human hepatocellular carcinoma cells *in vitro* as well as *in vivo*. Overall, aspirin shows great promise as a potent anticancer agent.
